# Successful preservation of the proximal stomach tube by evaluating blood flow using indocyanine green for gastric tube cancer: a case report

**DOI:** 10.1186/s40792-020-00848-3

**Published:** 2020-04-26

**Authors:** Kazushi Hara, Tomoyuki Matsunaga, Yoji Fukumoto, Wataru Miyauchi, Yusuke Kono, Yuji Shishido, Takehiko Hanaki, Kozo Miyatani, Joji Watanabe, Kyoichi Kihara, Manabu Yamamoto, Naruo Tokuyasu, Shuichi Takano, Teruhisa Sakamoto, Soichiro Honjo, Yoshiyuki Fujiwara

**Affiliations:** Division of Surgical Oncology, Department of Surgery, School of Medicine, Tottori University Faculty of Medicine, 36-1 Nishi-cho, Yonago, 683-8503 Japan

**Keywords:** Blood flow, Esophageal cancer, Indocyanine green, Intraoperative, Gastric tube cancer

## Abstract

**Background:**

There have been two reports on preserving the proximal gastric tube by using intraoperative indocyanine green (ICG)-based photodynamic detection to evaluate blood flow through the anastomosis for gastric tube cancer after esophagectomy. However, in those cases, the period since the first operation was > 3 years 11 months, and there have been no reports of cases with < 1-year periods after the first operation.

**Case presentation:**

A 59-year-old man underwent video-assisted thoracic subtotal esophagectomy and gastric tube reconstruction after two courses of preoperative chemotherapy for middle thoracic esophageal cancer. After half a year, follow-up upper gastrointestinal endoscopy showed a submucosal tumor in the posterior wall of the pre-pyloric region. We performed a biopsy, and the results led to a diagnosis of gastric cancer (moderately differentiated adenocarcinoma: tub2). Clinically, the patient was described as having stage IB (cT2N0M0) gastric cancer of the reconstructed gastric tube. To avoid total gastrectomy, we tried to evaluate the blood flow of the proximal part of the gastric tube by intraoperative ICG-based photodynamic detection. Intraoperative findings confirmed neo-vascularization from the remnant cervical esophagus to the upper region of the gastric tube approximately 7 cm through the esophagogastric anastomosis. Therefore, we dissected the distal part of the gastric tube approximately 4 cm from the esophagogastric anastomosis and then performed Roux-en-Y gastro-jejunostomy via the ante-sternum route. The postoperative course was stable, and the patient was discharged on the 14th postoperative day.

**Conclusions:**

ICG-based photodynamic diagnosis was found to be simple and less invasive. Therefore, even if the postoperative period is short, this method should be considered for evaluation of blood flow prior to performing less invasive surgery.

## Background

Gastric tube cancer, which occurs in the reconstructed gastric tube of patients after esophagectomy, is relatively rare, and it has been reported that the cumulative 10-year incidence of gastric tube cancer after esophageal cancer surgery was 8.6% [1]. Recently, the prognosis of esophageal cancer has been improving because of multidisciplinary treatment, including neoadjuvant chemotherapy and surgery, so the number of gastric tube cancer cases may increase in the future [2].

Standard treatment for gastric tube cancer after esophagectomy is considered to be the same as that for gastric cancer. In patients for whom endoscopic resection is impossible, total gastric tube resection with lymph node dissection is required, considering lymphadenectomy and anatomical blood flow. However, total resection of the gastric tube and jejunum reconstruction with super-charged vascular anastomosis is highly invasive and sometimes life threatening [3, 4]. The benefits of avoiding total gastric tube resection are that there is no need to touch the neck around the esophagogastric anastomosis where severe adhesion is thought to occur, and damage to surrounding organs, such as cervical vascularity and recurrent nerves, may be avoided. However, subtotal resection of the gastric tube that preserves the proximal part of the gastric tube is impossible, in anatomical theory, because the right gastroepiploic and right gastric arteries that supply blood flow to the reconstructed gastric tube should be dissected.

Indocyanine green (ICG) is a sterile anionic dye that binds to plasma proteins once injected into the vascular system via the intravenous route. On the basis of the properties of ICG, real-time intraoperative organ perfusion evaluation with ICG has been used in several clinical applications, including gastrointestinal surgery [5–7]. Recently, some reports have suggested that ICG methods could be used to preserve proximal lesions of the gastric tube, including the anastomosis site [8, 9].

In this report, we performed less invasive surgery for a patient with gastric tube cancer 10 months after esophagectomy. It is unclear how long it takes for neovascularization to develop through the esophagogastric anastomosis in the proximal region of the gastric tube after reconstruction. To the best of our knowledge, this is the first report of the ICG-based photodynamic method used to preserve the anastomosis site in gastric tube cancer ≤ 1 year after esophagectomy.

## Case presentation

**Fig. 1:**
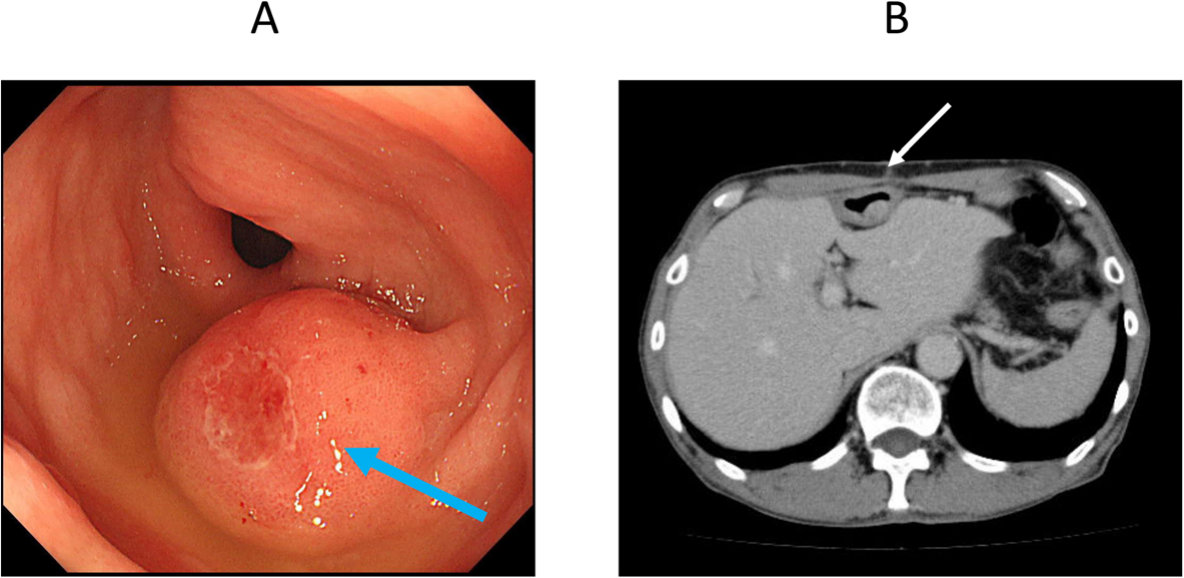
**a** EGD before chemotherapy. Submucosal tumor in the pyloric part of the gastric tube (blue arrow). EGD, esophagogastroduodenoscopy. **b** CT before chemotherapy. A 7-mm tumor (white arrow) located in the gastric tube. CT, computed tomography

**Fig. 2:**
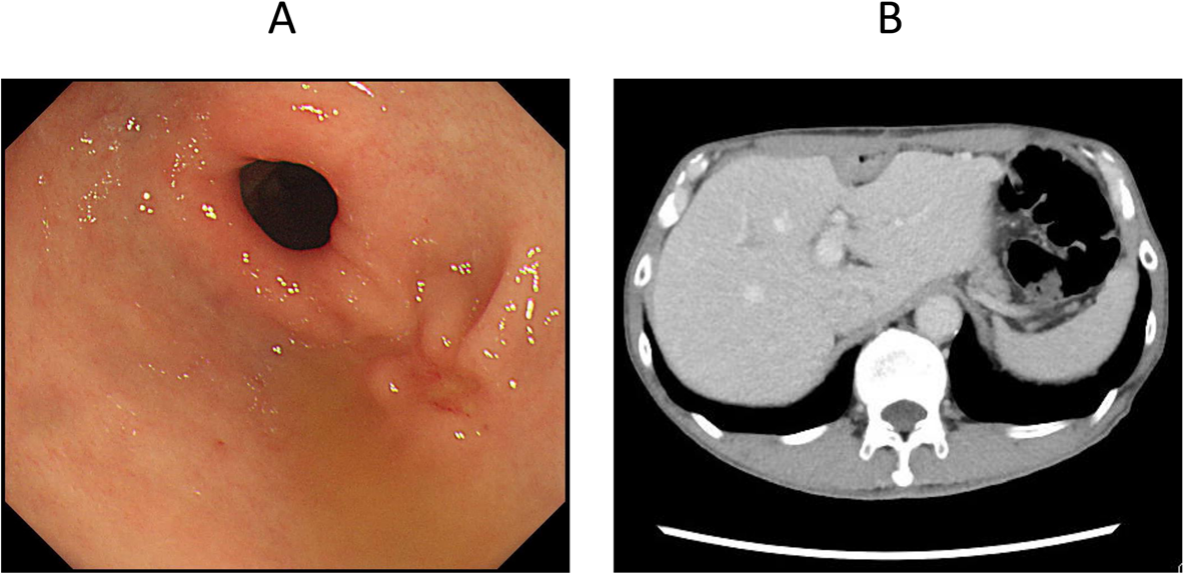
**a** EGD after chemotherapy. The tumor is significantly reduced and scarred. **b** CT after chemotherapy. The tumor is reduced and could not be clearly identified

**Fig. 3:**
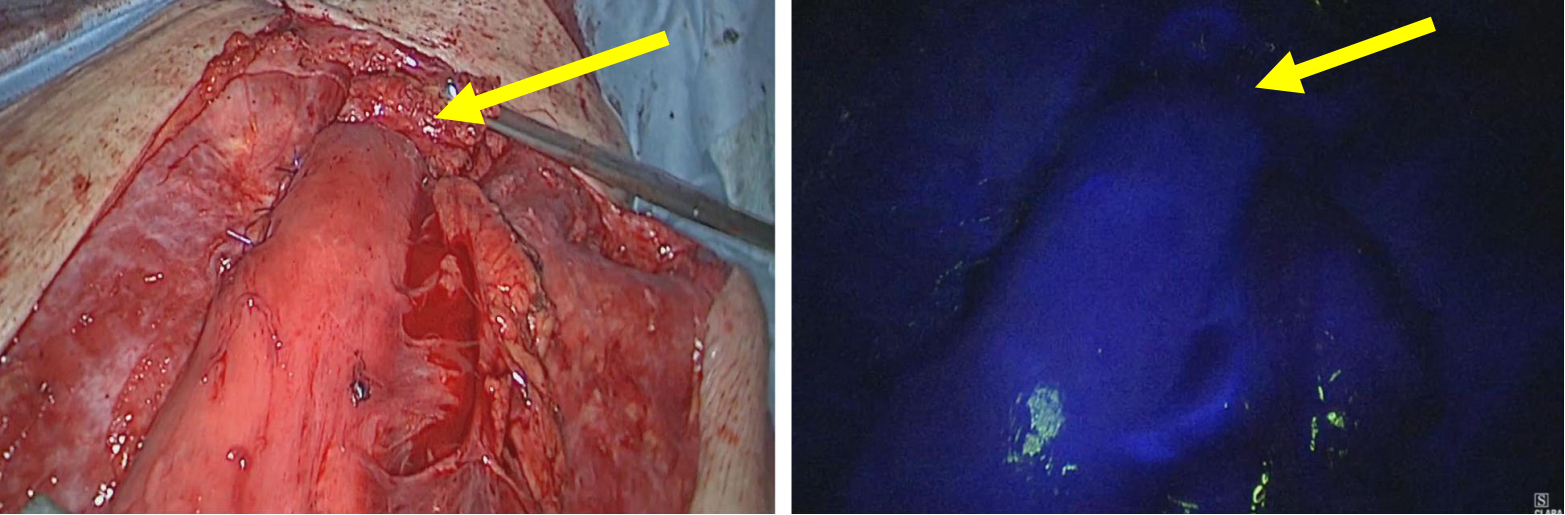
Intraoperative image. ICG 5 mg was injected after division of the right gastroepiploic artery and right gastric artery in addition to the duodenum 1 cm distal to the pylorus ring. There is sufficient inflow of ICG (blue indicates blood flow) from the esophagogastric anastomosis (yellow arrow) to the proximal gastric tube. ICG, indocyanine green

**Fig. 4:**
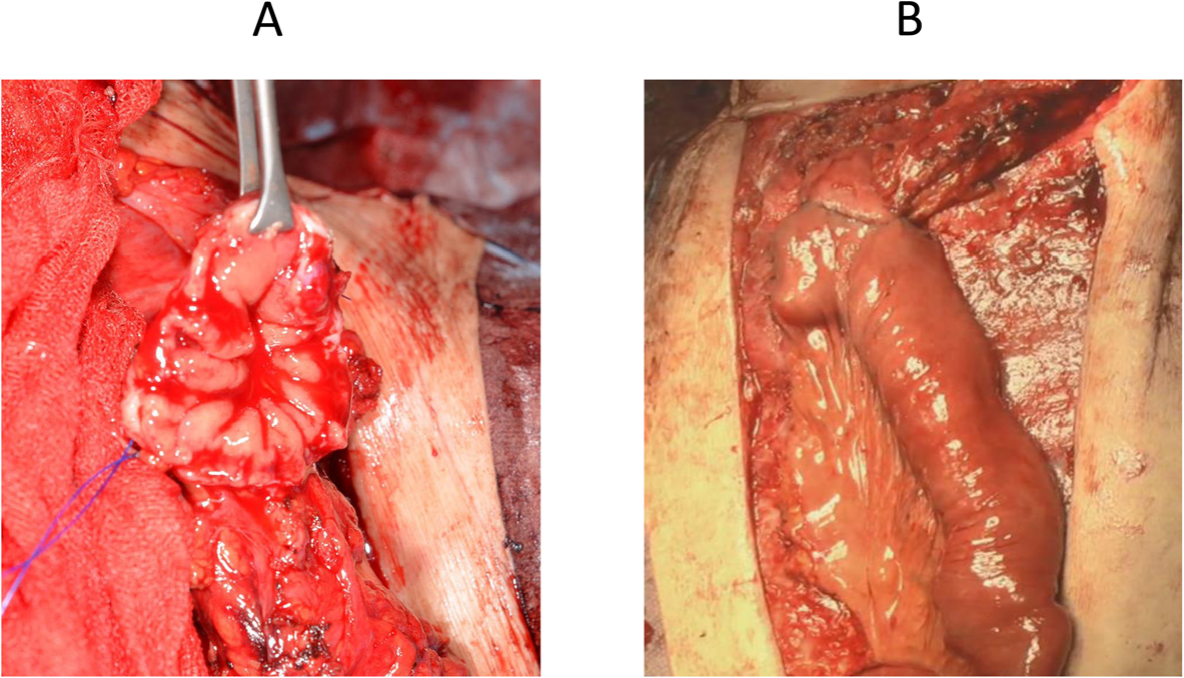
**a** The color of the cutting edge of the gastric tube is not pale, and bleeding is confirmed. **b** The completed anastomosis of the remnant gastric tube and jejunum

**Fig. 5:**
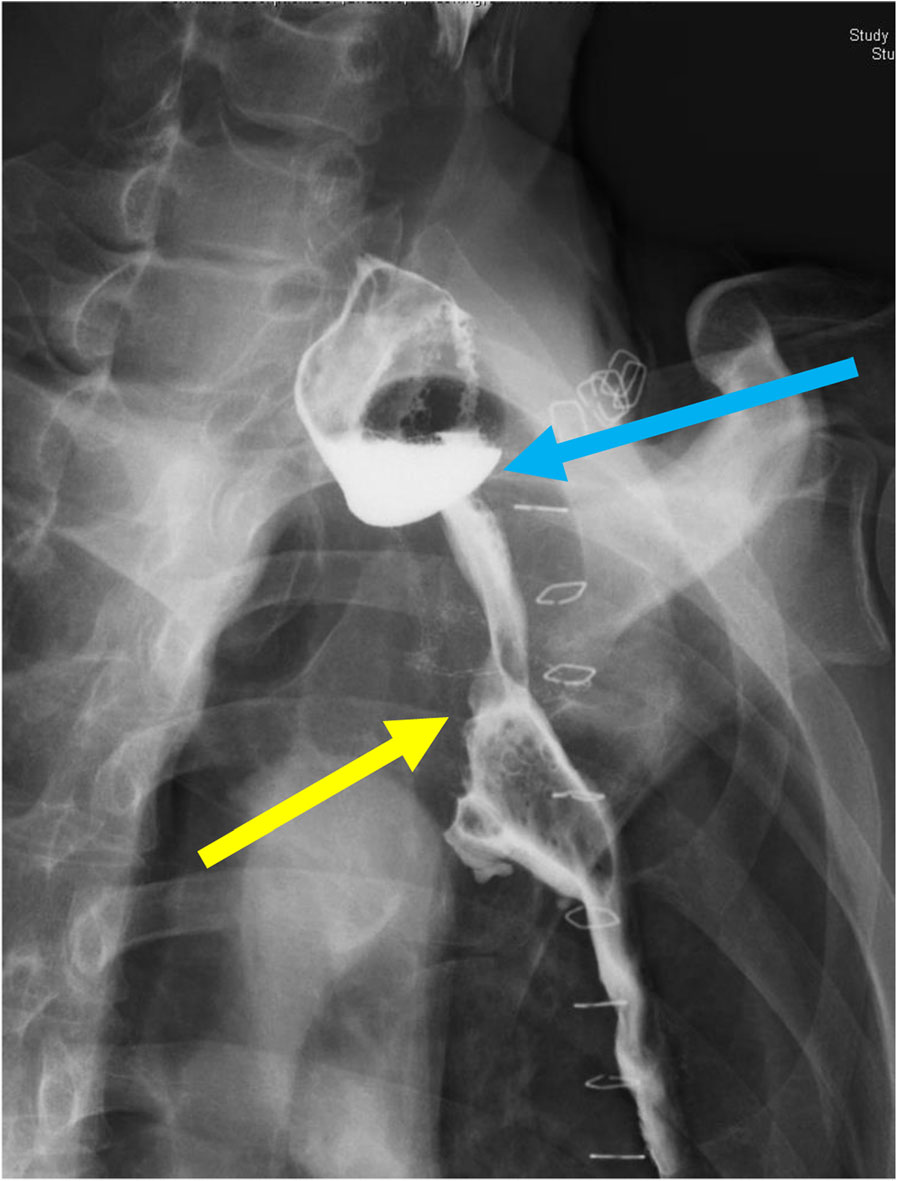
Image showing upper gastrointestinal fluoroscopy taken 8 days after the operation. The passage through both the esophagogastric tube anastomosis (blue arrow) and the gastric tube jejunal anastomosis (yellow allow) is good

A 59-year-old man was diagnosed as having middle thoracic esophageal cancer and had undergone video-assisted thoracic subtotal esophagectomy with gastric tube pull-up via the retro-sternal route at our hospital. The postoperative pathological result of esophageal cancer was stage II (pT1bN2M0). Although a submucosal tumor was observed in the gastric pyloric region before the operation, endoscopic ultrasound-guided fine-needle aspiration showed no malignant finding, so a decision to reconstruct the gastric tube was made. However, 6 months after surgery, follow-up upper gastrointestinal endoscopy revealed a tumor that was identified as a moderately differentiated adenocarcinoma (tub2) by biopsy. Computed tomography showed that there was no obvious lymph node metastasis or distant metastasis (Fig. 1), and the patient was diagnosed as having clinically stage IB (cT2N0M0) gastric cancer within the gastric tube. Because of patient’s request to perform the surgery 3 months later, we scheduled an operation after preoperative chemotherapy and two courses of S-1 + L-OHP therapy were performed. After preoperative chemotherapy, the size of the tumor had shrunk (Fig. 2). The operation was performed 10 months after the previous esophagectomy. First, we performed an upper midline abdominal incision so that the abdominal cavity could be observed. There was no obvious peritoneal dissemination or liver metastasis. After removing the adhesion around the front of the gastric tube, the right gastroepiploic artery and vein and the right gastric artery and vein were separated and ligated. Then, the duodenum was divided 1 cm distal to the pyloric ring by using an Endo GIA linear 45-mm stapling device with the Camel cartridge (Ethicon, NJ, USA). Next, we performed a midline sternotomy to expose the gastric tube, which was separated from the pleura, pericardium, sternum, thymus, and other organs. After exfoliation around the gastric tube, ICG 10 mg/2 mL was injected for blood flow evaluation via the peripheral blood route. The ICG fluorescence was illuminated by using a near-infrared laser beam with a laparoscopic system (KARL STORZ GmbH & Co. KG, Tuttlingen, Germany). Imaging was generated by using a high-end full high-definition camera system (IMAGE 1 SPIES™; KARL STORZ) connected to a laparoscope with a 30° field of direction and 10-mm diameter equipped with a specific filter. After injecting the ICG solution, blood flow was confirmed in about 20 s from the cervical esophagus to the upper region of the gastric tube approximately 7 cm through the esophagogastric anastomosis (Fig. 3). We judged that blood flow was good if it was confirmed blood flow within 30 s; hence, we judged that enough blood flow could be secured, and 4 cm of the proximal gastric stump region from the esophagogastric anastomosis was preserved. Reconstruction by performing a Roux-en-Y gastro-jejunostomy was accomplished via the ante-sternum route without vascular anastomosis (Fig. 4). The operation time was 359 min, and the blood loss was 560 mL. Pathological examination of a specimen identified pT1bN0M0 pStage IA gastric cancer (tub1). On postoperative day 8, X-ray examination of the upper gastrointestinal tract was performed; no stenosis of the anastomosis and remnant gastric tube was observed, and there was no suture failure of the anastomosis (Fig. 5). The postoperative course was stable, the patient was discharged on the 14th postoperative day, and he was doing well 15 months after surgery without recurrence.

## Discussion

Surgery is considered to be the only curative treatment for gastric tube cancer. However, total gastric tube resection is highly invasive and has risks for morbidity and mortality [4–10]. Some early gastric tube cancers can be managed with endoscopic treatment, because endoscopic submucosal dissection for gastric tube cancer is feasible and effective for curative patients [11]. In our case, we diagnosed the tumor as an advanced cancer, so we decided to perform surgical resection. Gastric tube cancer mostly occurs in the distal region of the gastric tube [12], and the tumor was located near the pyloric ring in our case. Regarding curability, it has been reported that total gastrectomy is necessary for upper gastric tube cancer, but that resection of the distal gastric tube would be sufficient for lower gastric tube cancer [13]. In our case, subtotal gastrectomy to preserve the proximal part of the gastric tube was considered to be feasible on the basis of the tumor’s location. The benefits of avoiding total gastrectomy are that it does not touch the esophagogastric anastomosis and shortens the distance of the pulled-up jejunum. In particular, total gastric tube resection requires vascular anastomosis to supercharge the jejunum blood flow at the time of reconstruction because the distance required to pull up the jejunum increases. Furthermore, a higher anastomotic site also disturbs the ability to swallow, which can lead to malnutrition and aspiration pneumonia. For these reasons, it is ideal to avoid total gastrectomy, so we used the ICG-guided photodynamic method to only resect the distal gastric tube without performing vascular anastomosis. In the case of insufficient blood flow, we planned to perform total gastrectomy and jejunum reconstruction with vascular anastomosis.

Damiano et al. retrospectively studied 202 patients with gastric tube cancer after esophagectomy [14]. They reported that in the majority of patients (*n* = 120), the posterior mediastinal route was used for reconstruction when esophageal resection was performed. The most difficult operative procedure of gastric tube cancer is the lysis of adhesion and removal of the gastric tube from the mediastinum. Thoracotomy is required for the removal of the gastric tube in the posterior mediastinum, and the total removal of the posterior mediastinal gastric tube is extremely difficult [15]. Therefore, this procedure that avoids total gastric tube resection is useful.

There have been two case reports, including three cases in which blood flow was assessed during surgery to help preserve the proximal part of the gastric tube during esophagogastric anastomosis for gastric tube cancer (Table 1) [8, 9]. In all of these cases, the proximal part of the gastric tube was successfully preserved by using ICG for blood flow evaluation during the operation after dissection of the right gastroepiploic artery and right gastric artery. In those cases, 5, 3.9, and 12 years had passed since the first operations, and it was considered that neovascularization was fully developed from the remaining esophagus via the anastomosis. In our case, distal gastrectomy was even successfully performed only 10 months after the first operation, which is very instructive information for surgeons interested in the treatment of esophageal cancer.

**Table 1:** Cases with gastric tube cancer treated by distal gastrectomy with ICG-guided photodynamic diagnosis

Age (years)	Sex	Location of esophageal cancer	Interval from esophagectomy (months)	Length of blood flow beyond esophagogastric anastomosis (cm)	Tumor location of gastric cancer	Preserved gastric tube length (cm)	Operative time and blood loss	pStage	POD of oral intake (days)	Hospital stay (days)	Reference number
52	M	Ut	67	5	Lower	3	538 min, 1490 mL	T2N0 Adeno Ca	14	25	8
50	M	Mt	47	5	Middle	3	783 min, 2855 mL	T4N0 SCC (recurrence)	31	62	8
69	F	Ae	154	Unknown	Middle	2	322 min, 370 mL	T2N0 Adeno Ca	7	28	9
59	M	Mt	10	7	Lower	4	359 min, 560 mL	T1bN0 Adeno Ca	8	19	Present case

*Ca* adenocarcinoma, *Ae* abdominal esophagus, *F* female, *GTC* gastric tube cancer, *ICG* indocyanine green, *M* male, *Mt* middle thoracic, *POD* postoperative day, *pStage* pathological stage, *SCC* squamous cell carcinoma, *Ut* upper thoracic

In the present case, the ICG photodynamic method was used to show that there was sufficient blood flow from the anastomosis, and the proximal gastric tube was preserved approximately 4 cm from the esophagogastric anastomosis. The length of the vascularized gastric tube varies among patients, so it is important to judge the cutting line where there is enough blood flow by performing intraoperative ICG evaluation. In previous reports, the preserved length of the proximal gastric tube was 3–5 cm [8, 9]. Therefore, it is speculated that at least 3 cm of the proximal gastric tube is vascularized and can be preserved.

## Conclusions

Although the number of cases was small and the accumulation of more cases is necessary, blood flow evaluation using the ICG-guided photodynamic method during surgery may be useful to preserve the proximal gastric tube even < 1 year after esophagectomy. This method is simple, not very invasive, and enables evaluation of blood flow in real time during surgery. Therefore, in patients with gastric tube cancer, less invasive surgery that avoids total gastric tube resection in combination with ICG-guided photodynamic blood evaluation should be considered even < 1 year after esophageal cancer surgery.

## Data Availability

All data regarding this paper are available on request.

## Abbreviations

ICG: Indocyanine green
